# Human Beta Cell Regenerative Drug Therapy for Diabetes: Past Achievements and Future Challenges

**DOI:** 10.3389/fendo.2021.671946

**Published:** 2021-07-16

**Authors:** Peng Wang, Esra Karakose, Lauryn Choleva, Kunal Kumar, Robert J. DeVita, Adolfo Garcia-Ocaña, Andrew F. Stewart

**Affiliations:** ^1^ The Diabetes Obesity Metabolism Institute, The Icahn School of Medicine at Mount Sinai, New York, NY, United States; ^2^ The Division of Pediatric Endocrinology, The Icahn School of Medicine at Mount Sinai, New York, NY, United States; ^3^ The Drug Discovery Institute, The Department of Pharmacological Sciences, The Icahn School of Medicine at Mount Sinai, New York, NY, United States

**Keywords:** human, diabetes, beta cell, insulin, regeneration

## Abstract

A quantitative deficiency of normally functioning insulin-producing pancreatic beta cells is a major contributor to all common forms of diabetes. This is the underlying premise for attempts to replace beta cells in people with diabetes by pancreas transplantation, pancreatic islet transplantation, and transplantation of beta cells or pancreatic islets derived from human stem cells. While progress is rapid and impressive in the beta cell replacement field, these approaches are expensive, and for transplant approaches, limited by donor organ availability. For these reasons, beta cell replacement will not likely become available to the hundreds of millions of people around the world with diabetes. Since the large majority of people with diabetes have some residual beta cells in their pancreata, an alternate approach to reversing diabetes would be developing pharmacologic approaches to induce these residual beta cells to regenerate and expand in a way that also permits normal function. Unfortunately, despite the broad availability of multiple classes of diabetes drugs in the current diabetes armamentarium, none has the ability to induce regeneration or expansion of human beta cells. Development of such drugs would be transformative for diabetes care around the world. This picture has begun to change. Over the past half-decade, a novel class of beta cell regenerative small molecules has emerged: the DYRK1A inhibitors. Their emergence has tremendous potential, but many areas of uncertainty and challenge remain. In this review, we summarize the accomplishments in the world of beta cell regenerative drug development and summarize areas in which most experts would agree. We also outline and summarize areas of disagreement or lack of unanimity, of controversy in the field, of obstacles to beta cell regeneration, and of challenges that will need to be overcome in order to establish human beta cell regenerative drug therapeutics as a clinically viable class of diabetes drugs.

## Introduction

Diabetes is a major global public health challenge. The World Health Organization estimates that there are 440 million people in the world with diabetes, the large majority of whom have Type 2 Diabetes (T2D) (1). Approximately 5% have Type 1 Diabetes (T1D). As summarized below, people with T1D, and most with T2D, suffer from reduced numbers of insulin-secreting pancreatic beta cells. Also, only ~30% of people with T1D or T2D achieve ADA therapeutic targets for glycemic control with currently available drugs. These considerations have prompted attempts to restore beta cell mass to normal in T1D through the use of whole pancreas transplant from organ donors, transplant of isolated human pancreatic islets, transplant of human embryonic or induced pluripotent stem cell-derived beta cells, and through the discovery of drugs that are able to induce human beta cells to replicate, proliferate or regenerate. This latter field of human beta cell regenerative drug development is moving rapidly. There is broad agreement that it is now possible to induce adult human beta cells to regenerate, but several areas of controversy as well as challenges remain. In this Review, we summarize recent advances and challenges in human beta cell regenerative drug therapy. In the first half, we focus on areas on which most investigators in the field agree. In the second half, we focus on areas in which there is room for disagreement, delineating unanswered questions and challenges in the field of human beta cell regenerative drug development.

## Areas of Consensus

### Beta Cell Mass Is Reduced In Diabetes

**Figure 1:**
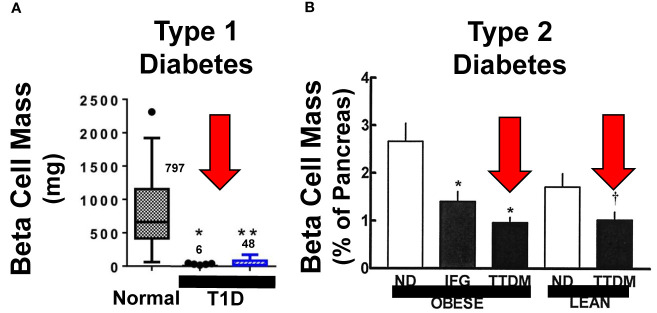
Beta Cell Mass in Type 1 and Type 2 Diabetes. **(A, B)** display beta cell mass assessed at autopsy in people with longstanding Type 1 diabetes (T1D) and Type 2 diabetes (T2D or TTDM), and people with impaired fasting glucose (IFG), also called metabolic syndrome. The red arrows highlight the relative reduction in beta cell mass in both T1D and T2D as compared to age, BMI and sex matched normal. Importantly, beta cells persist, in reduced numbers, in people who have had both T1D and T2D for decades, indicating that most people in both groups have residual beta cells that can serve as substrates for beta cell regenerative drugs. Adapted with permission from (2, 7). (2, 4–6) and (3, 7–9) provide additional examples. **(A)** Is reprinted with permission from (7). * and ** both indicate p < 0.001 vs. controls. The numbers within the graph indicate beta cell mass, in mg, in the three groups. **(B)** is reprinted with permission from (2). * indicates P < 0.01 and † indicates p < 0.05.

It seems clear that while insulin resistance is an important contributor to T2D, most people with obesity and insulin resistance do not have diabetes, suggesting that T2D results from a combination of insulin resistance together with another factor. Autopsy studies have reveal that pancreatic beta cell mass is reduced in some people with T2D by as much as 40-60% as compared to age-, sex- and body-mass index-matched normal, although there is substantial overlap with normals (**Figure 1**) (2, 4–6, 10). Understanding the timeline and cause of the reduced beta cell mass in T2D is difficult define in precise terms, since human beta cell mass can only be assessed at autopsy. The relative reduction in beta cell mass in many people with T2D likely reflects combinations of: 1) genetic predisposition to lower beta cell mass and/or reduced beta cell function, illustrated by GWAS studies (11, 12); 2) inadequate attainment of beta cell mass during fetal life and childhood (13, 14); 3) beta cell de-differentiation induced by glucotoxicity, lipotoxicity and/or endoplasmic reticulum (ER) stress, all driven by excessive demand for insulin resulting from insulin resistance and excessive caloric intake (15–18); and/or combinations of the above. Importantly in the current context, most people with T2D retain substantial beta cell mass, and most produce substantial quantities of insulin (2, 4–6, 10).

In T1D, beta cell mass is reduced as well, and is attributed to autoimmune destruction as well as de-differentiation of human beta cells (**Figure 1**). Based on autopsy studies, residual beta cell mass estimates in T1D, range from ~2% to 40% (3, 7, 8). Notably, most people with T1D, even after 50-80 years, continue to produce at least small amounts of insulin, and the large majority also have at least small numbers of residual beta cells at autopsy (8, 9).

These considerations underly the enthusiasm for beta cell replacement, which may be achieved by whole pancreas transplant (19, 20), by isolated human islet transplant (21, 22), or by transplant of stem cell-derived beta cells (23, 24) for people with diabetes. Of course, beta cell replacement will reverse T1D only if autoimmunity is managed through immunomodulatory drug or islet encapsulation approaches, both of which are currently imperfect, but making advances (18, 25). On the other hand, beta cell replacement for T2D is not widely performed, reflecting the facts that there is a broad range of drug therapies for T2D, and that beta cell replacement is simply impractical because of the sheer number of affected individuals. In addition, since beta cells are de-differentiated in T2D (10, 15–17), optimal drug therapies for T2D will need to induce human beta cells to re-differentiate from their de-differentiated state.

### The Definition of Authentic Beta Cell Proliferation

It is valuable to consider the types of assays for human beta cell replication. Typically, proliferation is assessed through immunocytochemical or immunohistochemical measurement of cell cycle or DNA synthesis markers in cells that co-immunolabel with insulin or c-peptide. These markers may include Ki67, BrdU, EdU, PCNA and phospho-histone H3 (PHH3), and occasionally others. BrdU, EdU and their analogues are thymidine analogues that are incorporated into DNA during S-phase of the cell cycle, and can be detected by immunolabeling with specific high-affinity antibodies. Using some of these markers (BrdU, EdU) requires that the cells, tissue or animals be pre-treated for hours or days before immunolabeling is performed. The percentage of cells labeled will be proportional to the number of days the cells or tissues are exposed to these chemicals, since BrdU and EdU remain incorporated into the DNA in the nuclei of the daughter cells after cell cycle is complete. Since they are mutagens, they cannot be used to label tissues in living humans. In contrast, Ki67, PCNA, and PHH3 can be assayed in dispersed islet cells and in tissue sections, and require no pre-labeling. Instead, they take advantage of the transient expression of these molecules during normal cell cycle progression, and their disappearance when cells complete cell cycle and return to quiescence.

Several points are important in interpreting these types of studies. First, although many authors, including ourselves, refer to the output of these studies in quantitative terms such as “replication rate”, or “proliferation rate”, they more accurately provide a “labeling index”, indicating the number of cells marked by Ki67, BrdU, etc., divided by the total number of cells immunolabeled for insulin. In human neonates, the beta cell labeling index for Ki67 is in the range of 2-3% (13, 26, 27). In normal adult human beta cells from autopsy or organ donors, the usual labeling index is in the range of 0.0-0.4% with Ki67, and similar for BrdU and EdU if tissues or cells are pre-labeled for 18-24 hours (13). Thus, if labeling is performed for 24 hours, and labeling index calculated 3 days later, “labelling index” will approximate a replication rate per 24 hours or “per day”. Conversely, if labeling is performed for 3-7 days, as is often done, labeling indices will be much higher. Thus, careful comparison of labeling time is important in studies of beta cell replication. As a correlation, one might assume that replication is cumulative, with exponential increases in beta cell mass over time. Unfortunately, for practical reasons, beta cell proliferation is typically measured at a single time point in most studies.

Second, immunolabeling for Ki67, PCNA, EdU, BrdU, etc., is not necessarily equivalent to actual cell division with the generation of daughter cells. For example, cells may initiate cell cycle entry, be labeled, but never complete the cell cycle, arresting in S-phase or G2M. Such cells will label for most of the cell cycle markers described above, giving a false impression of normal cell cycle completion. Analogously, since these same markers are activated in DNA damage and DNA repair pathways, expression of these “cell cycle markers” may instead reflect cell damage and impending death rather than true cell cycle completion. These events are commonly explored through immunolabeling with cell death markers or DNA damage markers such as TUNEL and γH2AX, respectively (15).

Third, while markers of proliferation as described above are useful for rapid and high-throughput assessment and quantification of proliferation, the gold standard for unequivocal generation of new beta cells should, of course, be documentation of an increase in the actual number of beta cells. We have approached this goal in two ways. In one, we have used an adenovirus that delivers a fluorescent protein called ZsGreen in a beta cell-specific manner using the Rat Insulin 1 Promoter (RIP1) to human islets. In a second, we used a human ES cell line in which GFP has been knocked-into the one allele of the insulin locus. These maneuvers enabled beta cell labeling and quantification by flow cytometry (28, 29). In each case, as described below, drug treatment led to a clear, statistically and quantitatively significant increase in the number of human beta cells as will be discussed in detail below. Thus, this technique confirms the ability of at least some drugs to expand adult human beta cell numbers and mass.

### Rodent *vs*. Human Beta Cell Replication

**Figure 2:**
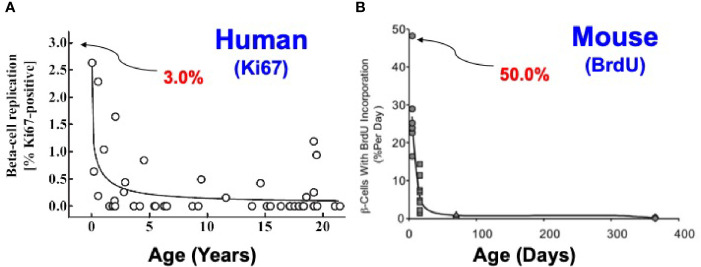
Comparison of Beta Cell Proliferation Rates as a Function of Age in Humans and Mice. The key point is that beta cells replicate at high rates in juvenile humans and mice, and decline with age. With age (early adult hood in humans and ~12 months in mice), beta cell replication ceases and is difficult or impossible to re-activate in both species. Note that at birth, beta cell proliferation is much higher in rodents than in humans. Note also that most rodent studies employ animals that are 2-3 months of age, whereas most human studies employ islets from organ donors who are 40-55 y.o. Similar studies are available in (27, 30, 31). **(A)** Is reprinted with permission from (26). **(B)** Is reprinted with permission from (32).

Rodent models have been used extensively to assess beta cell proliferation. At birth, beta cell replication rates (labeling indices) in mice are high (20-30%), and decline over time, such that they are low (<1%) by 12-18 months of age, and are difficult or impossible to activate (**Figure 2**) (30, 32). Many maneuvers in rodents, such as high fat feeding, glucose infusion, partial pancreatectomy, beta cell ablation with streptozotocin or diphtheria toxin, or drug treatment, for example with GLP1 receptor agonists such as exendin-4, are followed by a rapid increase in beta cell proliferation (reviewed in 30, 32 and “Candidate Drugs” below). Most often, however, these studies are performed in mice that are 2-4 months old. Notably, when similar studies performed in older mice, >1 year of age, there is little proliferative response to the same maneuvers (30). Thus, in juvenile rodents, beta cells display regenerative capacity, but this plasticity disappears with age. Of course, since purchasing and maintaining rodent colonies is expensive, pressure to complete studies and costs dictates that most studies in “adult” rodents are performed in 2-4 month-old rats and mice.

In humans, similar dynamics apply, with two important differences. Beta cell proliferation increases in the first year of life, peaking at 2-3% (far lower than rodents) and then declines through childhood to rates that are low or undetectable in adults (**Figure 2**) (13, 26, 27). Most human islet studies are performed in islets recovered from adult organ donors, in the 40-60 y.o. range, and do not proliferate appreciably in response to high glucose, growth factors, hormones or drugs. They compare in replication rates to rodents 12-18 months of age. Thus, beta cells from both rodents and humans display their highest rates of replication shortly after birth, but the rates in the early postpartum period are much higher in rodents. Interestingly, beta cells from fully adult rodents and humans are recalcitrant to replication. Again, for practical reasons, most beta cell replication studies are performed in juvenile rodents, but in islets from fully adult humans. There is a middle ground in humans. In rare studies where juvenile human islets have been studied, replication can be induced by glucose stimulation or GLP1 receptor agonists such as exendin-4 (33, 34). Of course, since any future intended therapeutic drug treatment will first be evaluated in adults, and since only adult islets are available in reliable quantities, induction of proliferation in adult human beta cells represents the key hurdle for human studies. We discuss the underlying mechanisms for recalcitrance of adult beta cells to replication in the second half of this review.

### Candidate Drugs and Other Molecules for Human Beta Cell Proliferation

Because of the urgent need for beta cell regenerative drugs, many laboratories in academia and industry have explored the discovery and development of beta cell mitogenic nutrients, growth factors and drugs, commonly using juvenile rodent islets as a model system. Examples of agents proposed to induce beta cell proliferation are many, and include glucose (33), lactogens such as placental lactogen or prolactin (35), growth hormone (36), PTH-related protein (PTHrP) (37), hepatocyte growth factor (HGF) (38), dextran sulfate (39), the short peptide TLQP21 (40), serpin B1 (41), GLP1 family peptides exemplified by exendin-4 (34), gamma amino-butyric acid (GABA) (42), purinergic agonists and adenosine kinase inhibitors (43, 44), TGFβ inhibitors (45), and inhibitors of glycogen synthase kinase 3β (GSK3β) (46) and of dual specificity tyrosine-regulated kinase 1A (DYRK1A). Most of these do reproducibly induce beta cell replication in juvenile rat or mouse beta cells *in vitro* and/or *in vivo*. But among this group, only DYRK1A inhibitors have been shown reproducibly increase replication of human beta cells at rates in excess of 1%. Therefore, in this review, we focus on DYRK1A inhibitors.

### DYRK1A Inhibitors Induce Adult Human Beta Cells to Replicate

**Figure 3:**
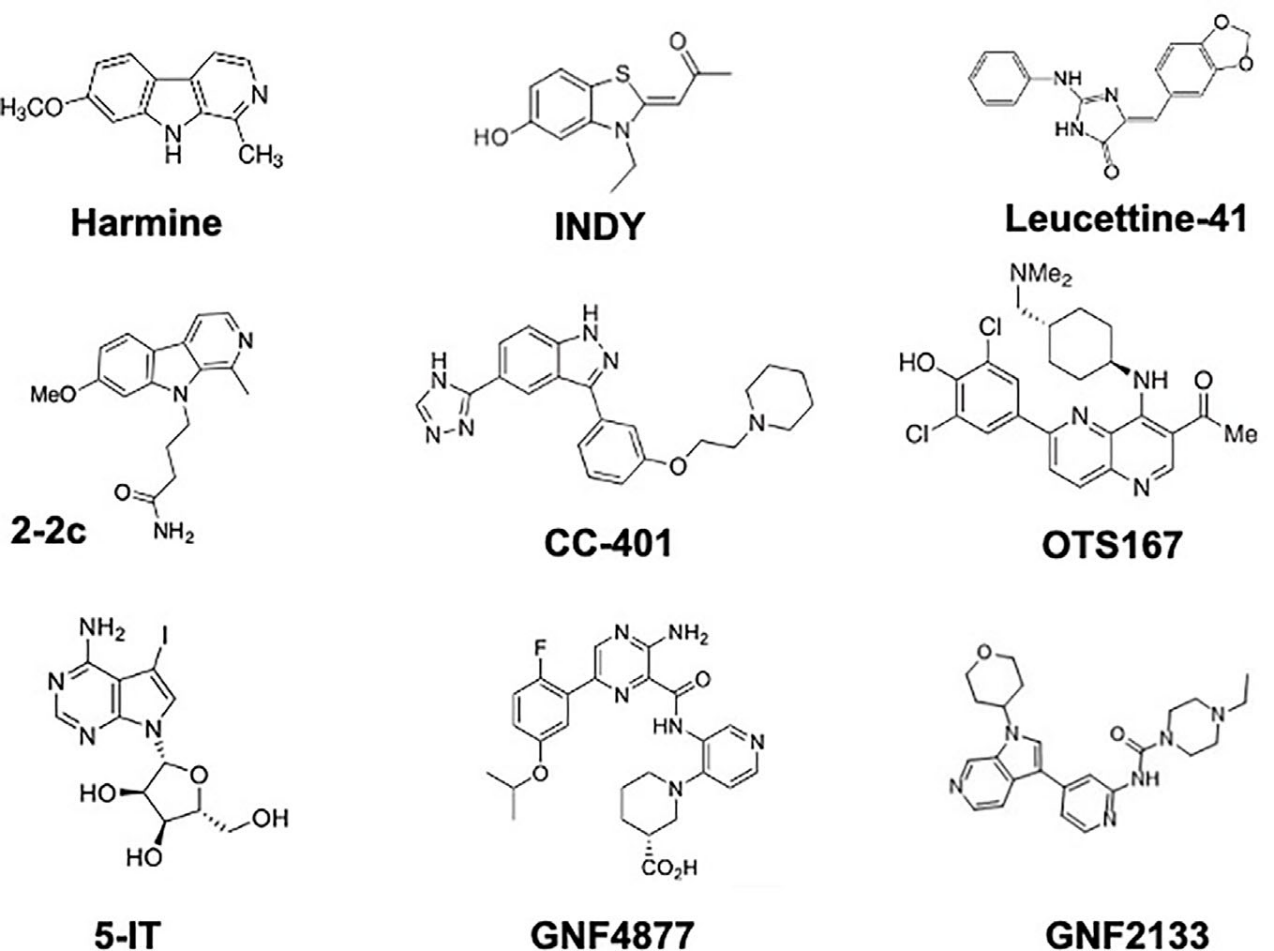
Chemical Structures of DYRK1A Inhibitors That Have Been Demonstrated to Induce Human Beta Cell Proliferation. See text for details and references.

Multiple members of the DYRK1A family are illustrated in **Figure 3**. The first report of a DYRK1A inhibitor able to induce beta cell proliferation was reported in 2012 by Annes et al, who demonstrated that 5-iodotubericidin (5-IT) is able to induce rodent and porcine beta cells to replicate, an effect initially attributed to the ability of 5-IT to inhibit adenosine kinase (47). In 2015 through 2020, multiple groups including Laffite et al., Wagner et al., Annes et al., and ourselves showed that multiple DYRK1A inhibitors - harmine, INDY, leucettine-41, GNF4877, GNF2133, CC-401, OTS-167, and 2-2c - are able to induce human beta cells to replicate, as assessed by Ki67, BrdU, EdU, PHH3 immunolabeling, at rates of 2-3% (28, 29, 47–55). Importantly, human beta cell proliferation can be reproduced by directly silencing DYRK1A gene expression in human islets (28, 29, 48–51). Conversely, proliferation in response to DYRK1A inhibitors can be blocked by overexpression of DYRK1A in human islets (28, 29, 48). Moreover, since no small molecule inhibitor of DYRK1A is entirely specific for DYRK1A (all inhibit other structurally related kinases such as DYRK1B, DYRK2, DYRK3, DYRK4, the CLK1-4 family, GSK3α and GSK3β), we genetically silenced these potential additional targets in human islets and observed no human beta cell proliferation (51).

### DYRK1A Inhibitor Mechanism of Action

DYRK1A is a kinase that phosphorylates a number of substrate proteins, among which are the Nuclear Factor activated in T-cells (NFaT) family of four transcription factors (31, 48, 55–57). NFaTs normally reside in the cytoplasm, in a phosphorylated state. Upon calcium entry into beta cells, for example in response to glucose, sulfonylureas, or GLP1 receptor agonists, calmodulin is activated, and in turn activates the phosphatase calcineurin. Calcineurin dephosphorylates NFaTs, allowing them to enter the nucleus, where they bind to regulatory regions of target genes, activating genes encoding cyclins and cdks (e.g., cdk1, cyclin A, cyclin E) and repressing genes encoding cell cycle inhibitors (e.g., p57^KIP2^, p15^INK4^), the net effect of which is activation of beta cell proliferation. The role of DYRK1A in this process is to re-phosphorylate nuclear NFaTs, forcing their nuclear exit, thereby terminating their mitogenic signaling. Thus, one can think of DYRK1A as being a “brake” on beta cell proliferation, and DYRK1A inhibitors as “removing the brakes” on beta cell proliferation. While this scenario is sound and well supported, additional DYRK1A pathways and targets exist, and may also participate in driving human beta cell proliferation. These are discussed in the second portion of this review on Challenges and Controversies.

### DYRK1A and DYRK1B Inhibitor Combinations

In parallel studies, we noted that silencing DYRK1A led to markedly increased expression of DYRK1B at the protein, but not mRNA, level (51). We silenced DYRK1B in human beta cells, but noted no effect on human beta cell proliferation. Importantly, however, silencing DYRK1A and DYRK1B simultaneously led to a synergistic effect on human beta cell proliferation. Thus, since all DYRK1A inhibitors are also DYRK1B inhibitors, it appears that these drugs fortuitously inhibit two separate inhibitors of human beta cell proliferation. It is unknown at present exactly how DYRK1B inhibition operates to facilitate DYRK1A inhibition-induced proliferation. Hereafter, we refer to the drugs in this class as “DYRK1A inhibitors”, but it is important to be clear that all are also DYRK1B inhibitors.

### DYRK1A Inhibitor Proliferative Efficacy

**Figure 4:**
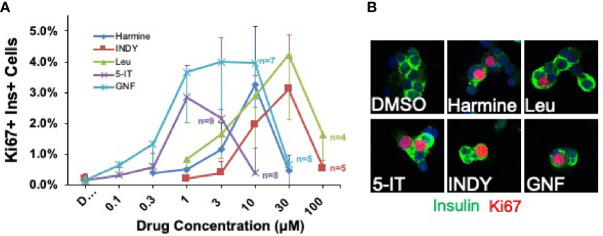
Head-to-Head Comparison of Lead Beta Cell Regenerative Drugs. **(A)** Five most widely studied DYRK1A inhibitors were directly compared in the same cadaveric donor islets. Each line represents a dose-response curve in the same islet preparations assayed at the same time at the concentrations shown. The same 10 adult human islet preparations were used for all data points, except in cases where sufficient human islets were not available to assay all drugs at every dose: in these cases, the number of human islet preparations used at a given dose is indicated. Several points are important. First, there typically is great variability among human islet preparations, as illustrated here by the large error bars (SEM). Second, GNF4877 and 5-IT are the most potent in driving human beta cell proliferation, followed by harmine and leucettine and INDY. Third, the maximum efficacy in inducing proliferation is similar for all DYRK1A inhibitors. Fourth, all compounds inhibit human beta cell proliferation at higher doses, suggesting that they interfere, at higher doses, with important cell cycle activation pathways. Compare to **Figure 10** below. **(B)** Both panels are adapted with permission from (51), where statistical details and findings with additional DYRK1A inhibitors can be found.

Each of the DYRK1A inhibitors described above is able to induce human beta cells to replicate, and peak beta cell proliferation rates are comparable. The maximal rate is dependent on the human islet donors and batches. Accordingly, we directly compared the ability of harmine, GNF4877, 5-IT, leucettine-41 and CC-401 to induce human beta cell Ki67 immunolabeling in the same 10 batches of human islets (**Figure 4**) (51). The two most potent were GNF4877 and 5-IT (EC_50_ for both ~0.5 µM), followed by harmine and leucettine-41 (both ~5 µM), then INDY (~10 µM). The maximal labeling index for all compounds was similar (3-4% in these batches of islets). Of note here is the fact that every DYRK1A inhibitor studied actually inhibits proliferation at higher doses, suggesting that at such doses, they interfere with additional molecules and pathways required for proliferation.

### DYRK1A Inhibitor Promiscuity *vs*. Specificity

As noted above, DYRK1A inhibitor drugs are not exclusive inhibitors of DYRK1A: they inhibit additional kinases. This reflects the fact that the active site in DYRK1A through which these drugs act binds ATP, required for phosphorylation of DYRK1A substrates. Thus, many additional kinases, all of which bind the ATP site, are targeted by harmine, INDY, 5-IT, GNF4877, etc. Examples include all of the DYRK family members, CDC-like kinases (CLKs), GSK3 kinases (49–53). For example, in a screen against 468 human kinases at 10 uM, we found that GNF4877 interacted with (selectivity score <10) 103 kinases, 5-IT with 92 kinases, harmine with 10 kinases and the harmine analogue 2-2c with 6 kinases (51, 52). The implications of these observations are several, including the concepts that harmine and 2-2 are relatively “clean” and therefore predicted to have lower risks of adverse effects as compared to compounds that hit multiple alternate kinase targets. Accordingly, a goal of future DYRK1A inhibitor design, as discussed in Challenges and Controversies below, is to reduce interactions with kinases other than DYRK1A while enhancing potency of DYRK1A inhibition, as well as identifying additional kinase targets (if any) which may further enhance or synergize with DYRK1A inhibition to augment human beta cell proliferation.

### DYRK1A Inhibitors in Combination With TGFβ Superfamily Inhibitors

While DYRK1A inhibitors generate human beta cell proliferation rates (labeling indices) in the 2-4% range, one can envision a need for higher rates of proliferation, especially in people with T1D who have particularly low numbers of residual beta cells. Accordingly, we investigated combinations of DYRK1A inhibitors with other drugs that might synergize to yield higher rates of human beta cell proliferation. We elected to explore TGFβ superfamily inhibitors because several other labs had found that TGFβ inhibition activates rodent beta cell proliferation (45, 58, 59), and because we observed abnormalities in DYRK1A gene expression as well as abundant mutations in SMAD pathway genes in the proliferating beta cells of insulinomas (60). We found that TGFβ inhibitors had little effect on human beta cell proliferation when used alone (28). In contrast, combining TGFβ inhibitors with harmine, INDY or leucettine led to a dramatic increase in adult human beta cell proliferation, with labeling indices for harmine alone of 2-3%, and for harmine plus a TGFβ inhibitor averaging 5-8%, and in some islet donors rose to 15-18% (28). Annes et al. observed similar synergy using the DYRK1A inhibitor, CC-401, and the TGFβ inhibitor, ALK5 inhibitor (53). This synergy required inhibition of DYRK1A, was associated with interruption of SMAD signaling, and could be mimicked by simultaneous genetic silencing DYRK1A and SMAD family members (28). The high rates of proliferation suggested that it may be possible to actually observe an increase in human beta cell numbers, despite the brief survival of cultured human beta cells. To assess this, as described above, we employed a rat insulin promoter-1 (RIP1)-driven adenovirus expressing the bright green fluorescent protein, ZsGreen, to selectively label beta cells, and then quantified human beta cell numbers by flow cytometry in human islets exposed to vehicle or to the harmine plus TGFβ inhibitor combination. The harmine plus TGFβ inhibitor combination led to a near-doubling of human beta cell numbers in only four days (28). These results were independently corroborated in human stem cell-derived beta cells in which green fluorescent protein (GFP) was knocked into one allele of the insulin locus (28). This constituted the first evidence that any putative human beta cell mitogenic drug could increase actual human beta cell numbers, as compared to merely increasing beta cell proliferation markers.

### DYRK1A Inhibitors in Combination With GLP1 Receptor Agonists

**Figure 5:**
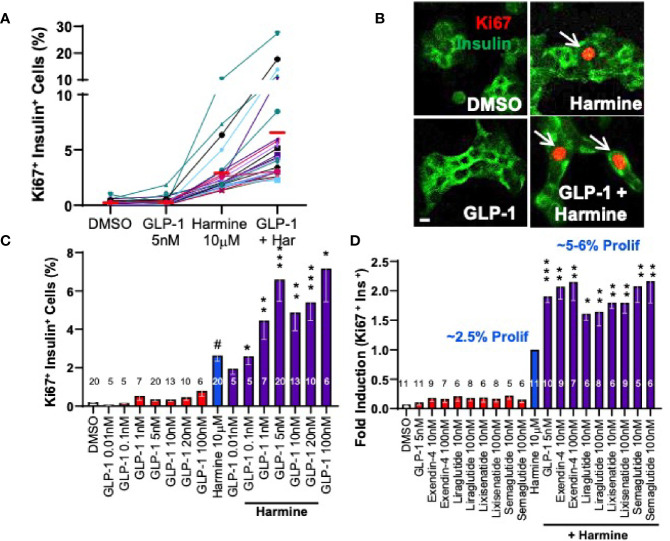
Adult Human Beta Cell Proliferation in Response to Combined Treatment with Harmine and GLP1R Agonists. **(A)** Proliferation (Ki67 labeling index) in human islets exposed for 72 hours to vehicle (0.1% DMSO), GLP1, harmine or GLP1 plus harmine. Each colored line represents one of 20 different human islet preparations assayed for all four treatments. The red bars represent the mean for each dose. **(B)** An example of Ki67 immunolabeling (red) in dispersed human beta cells (green). **(C)** Dose-response curves for Ki67 labeling in human beta cells in response to multiple doses of GLP1 alone, or the maximally effective dose of harmine (10 µM) alone, or the harmine-GLP1 combination. **(D)** Comparison of clinically available GLP1 receptor agonists alone or in combination with harmine. Note that for harmine, Ki67 labeling is 2.4%, but is normalized to 1.0-fold, so that the 2-fold increase in proliferation for the combinations reflects mean Ki67 labeling indices of 5-6%. Note also that none of the GLP1R agonists in clinical use induce human beta cell proliferation. All panels reproduced with permission from (29), which contains complete details. *p < 0.05; **p < 0.01, ***p < 0.001.

**Figure 6:**
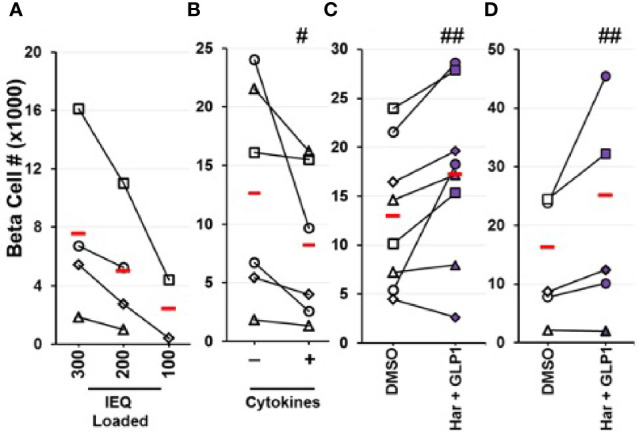
Quantification of Changes in Actual Human Beta Cell Numbers by Flow Cytometry. **(A–C)** Human islet cells were dispersed, beta cells labeled with an adenovirus expressing the bright green fluorescent protein, ZsGreen, under the control of the rat insulin-1 promoter, followed by flow cytometry to quantify green cells (28). **(A)** Represents a negative control: progressively decreasing numbers of human islets were labeled, then counted by flow cytometry. As expected, as fewer human islets were labeled, fewer beta cells were counted, in a dose-related manner. **(B)** Shows a second negative control: human islets were labeled with RIP1-ZsGreen adenovirus, and treated with vehicle or cytokines (IL-1β, TNFα) that induce human beta cell death. Again, as expected, reduced numbers of beta cells were counted. In **(C)**, human islets were treated with vehicle (DMSO) or the harmine-GLP1 combination, then labeled with ZsGreen as in prior panels. This resulted in an increase in beta cell numbers in 7 of 8 human islet preparations averaging 40% over four days of treatment. **(D)** Similar studies in human embryonic stem cell-derived beta cells, in which green fluorescent protein had been inserted into the insulin gene locus in one allele, permitting visualization and counting of beta cells, without employing adenovirus exposure. Again, treatment with vehicle (DMSO) or the harmine-GLP1 combination led to greater hESC-derived beta cell numbers in four of five preparations, averaging 50% higher over seven days. Reproduced with permission from (29), which contains complete details. ^#^p < 0.001, ^##^p < 0.01.

Concerned that TGFβ inhibitors would have undesirable off-target effects on the broad range of known TGFβ inhibitor target tissues, we sought alternate potential synergistic partners for DYRK1A inhibitors that might add a degree of beta cell specificity. We explored a variety of compounds active in beta cells, including meglitinide drugs and sulfonylurea drugs that target the SUR1/Kir6.2 potassium channel on beta cells, and the glucagon-like peptide (GLP1) family that target the GLP1 receptor (GLP1R) on beta cells. All three classes of drugs are in widespread clinical use for diabetes because of their ability to augment insulin secretion in people with diabetes. We found that meglitinides and sulfonylureas had no human beta cell mitogenic activity on their own, and did not synergize with harmine. In contrast, although GLP1 family drugs had no intrinsic beta cell mitogenic activity in adult beta cells, as is well described (34, 53), they markedly enhanced the ability of harmine to drive adult beta cell proliferation (Ki67 labeling indices), from the usual 2-3%, to 5-7% on average, and in some islet donors, increasing to 20-30% (**Figure 5**) (29). This synergistic increase in human beta cell proliferation was a class effect for all DYRK1A inhibitors studied and for all GLP1 analogues studied, including many in current clinical use, exemplified by exenatide, liraglutide, lixisenatide and semaglutide (29). Finally, we used our flow cytometric assay described in the preceding section to quantify human beta cell numbers in response to treatment with vehicle alone *vs*. harmine plus GLP1 (**Figure 6**). These studies demonstrated that actual human beta cell numbers increased from 12,000 to 17,000 in only four days of treatment (29). Similarly, human ES-cell -derived beta cells increased from 13,000 to 21,000 in only 7 days of treatment (29). Thus, as with the harmine-TGFβ superfamily inhibitor combination, the harmine-exenatide combination increases not only markers of human beta cell proliferation, but also increases actual numbers of human beta cells.

We found that the harmine-GLP1 synergy required inhibition of DYRK1A: harmine could be replaced by genetic silencing of DYRK1A; conversely, DYRK1A overexpression blocked the synergistic proliferation (29). The synergy also required activation of cAMP signaling: GLP1 could be replaced by any agent that increased beta cell cAMP concentrations, and the synergy could be blocked by inhibitors signaling downstream of cAMP, such as protein kinase A and EPAC2 (29).

**Figure 7:**
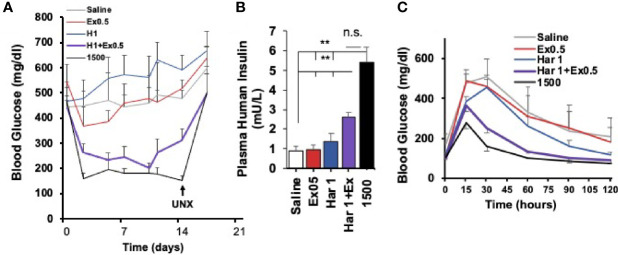
Effects of The Harmine-GLP1 Combination on Human Beta Cell Function *In Vivo*. **(A)** Using a standard marginal mass human islet transplant model in streptozotocin (STX) diabetic mice, 500 human islets were transplanted into the renal capsule of immunodeficient mice, which were then treated intraperitoneally for two weeks with saline, harmine (1 mg/kg/d), the stable GLP1 analogue, exendin-4 (0.5 µg/kg/d) or the harmine-exendin-4 combination. Mice transplanted with 1500 human islets served as a positive control. Note that saline, harmine alone or exendin-4 alone had little effect on blood glucose in the diabetic mice, but the harmine-exendin-4 treatment resulted in a marked improvement, approaching the efficacy of the 1500 islet group. Diabetes recurred following unilateral nephrectomy (UNX) of the human islet graft, confirming that glycemic control resulted from enhanced function of the human graft, and not from endogenous mouse beta cells. **(B)** Human insulin measurements in the five groups on day 14. Note that human insulin concentrations were three times higher in harmine + exendin-4 group than the harmine or exendin-4 alone groups, but not as high as in the mice receiving 1500 human islets. **(C)** Intraperitoneal glucose tolerance tests at day 14 in the groups in **(A)** Glycemic control is much improved in the animals receiving the harmine-exendin-4 combination as compared to the saline, harmine only, or exendin-4 only groups, approaching the 1500 islet positive control group. Reproduced with permission from (29), which contains complete details. **p < 0.01; ns, not significant.

Importantly, the harmine-GLP1 synergy was operative *in vivo* in human islets transplanted into immunocompromised mice (**Figure 7**). For example, diabetic mice transplanted with a marginal human islet number (500 islet equivalents, IEQ), an islet number inadequate to reverse their diabetes, remained diabetic for the full two weeks of the study, and the same was true for identical mice treated with exenatide alone or harmine alone (29). In contrast, mice treated with the harmine-exenatide combination rapidly corrected their hyperglycemia to near normal levels, and increased their circulating levels of human insulin. Moreover, they also tripled the rate of human beta cell proliferation *in vivo* in the human islet grafts.

### Harmine Induces Human Beta Cell Differentiation

**Figure 8:**
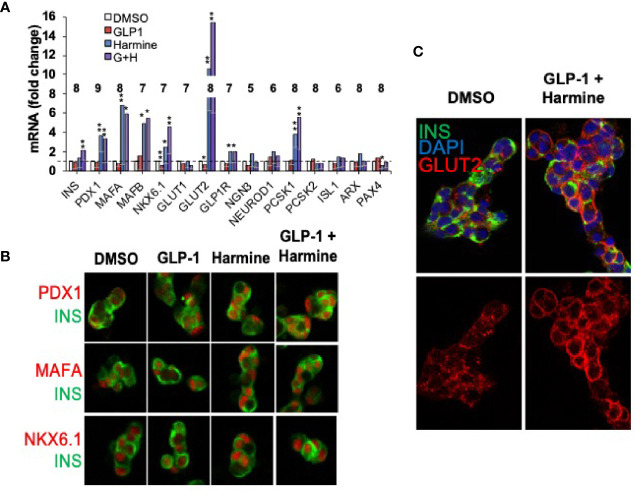
Harmine Alone and In Combination With GLP1 Enhances Human Beta Cell Differentiation. **(A)** Q-PCR gene expression studies on human islets treated with vehicle (DMSO), harmine alone, GLP1 alone or the combination. Note that harmine alone and in combination with GLP1, increases expression of canonical beta cell transcription factors and differentiation markers. The numbers within the bars represent the number of different human islet batches tested. **(B)** Immunocytochemistry showing that key beta cell transcription factors (red) are present, as expected, in human beta cells (green), and increase in abundance following harmine or harmine-GLP1 treatment. **(C)** The glucose transporter, GLUT2, increases in abundance in human beta cells and appears in the cell membrane with the harmine-GLP1 combination. Reproduced with permission from (29), which contains complete details. *p < 0.05, **p < 0.01.

The preceding sections have focused on human beta cell proliferation. As noted earlier, however, in addition to reduced numbers of beta cells in T2D and T1D, beta cells lose differentiation in T1D and T2D (16–18). Accordingly, attempts at beta cell regeneration would benefit from drugs that maintain or enhance human beta cell differentiation. Against this background, we had assumed that activating proliferative pathways might lead to beta cell de-differentiation, a concern that led us to explore beta cell differentiation status in human beta cells induced to replicate in response to harmine alone or in combination with TGFβ superfamily inhibitors or with exenatide. Remarkably, and serendipitously, we found that harmine alone or in combination with TGFβ superfamily inhibitors or with exenatide actually enhanced expression of canonical markers of beta cell differentiation {insulin, PDX1, MAFA, NKX6.1, SLC2A2 (encoding the GLUT2 glucose transporter), GLP1R, PCSK1, etc.}, at the mRNA and protein levels, and enhanced glucose-stimulated insulin secretion from human islets (**Figure 8**) (28, 29, 48). These effects were also observed in beta cells derived from organ donors with T2D (29). Remarkably, in each study, these effects could be attributed to harmine, and were not affected by addition of TGFβ superfamily inhibitors or by exenatide (28, 29, 48). By way of independent confirmation, Dirice et al. also noted that 5-IT induces expression of *SLC2A2*, the gene encoding GLUT2 (50). The cellular mechanism of action for the beneficial effects of harmine on beta cell differentiation is unknown.

## Pressing Challenges and Controversies

The brief summary above, all defined in the last 5 years, is widely accepted as valid, at least in a general sense, in the beta cell research community. There are many unanswered and controversial issues that have now moved to the forefront of interest, and in many cases, to controversy, in the field of human beta cell regeneration for T2D. In the sections that follow, we provide an overview of several areas of scientific challenge and/or controversy.

### Is The Reported Human Beta Cell Proliferation Authentic?

As noted above, increases in markers of proliferation, such as Ki67, PCNA, PHH3, BrdU or EdU labeling do not unequivocally prove that authentic proliferation - completion of cell division with the generation of two daughter cells - has occurred. As noted earlier, these same molecules can be involved in DNA repair and early cell death. Thus, it has become customary in beta cell proliferation studies to assess measures of cell death and DNA damage (TUNEL labeling, γH2AX immunolabeling) to assure that these latter two activities do not account for observed increases in proliferation markers. This practice makes it unlikely that authentic cell cycle entry does not occur in response to DYRK1A inhibitors. For those interested in more details, Alonso et al. have provided convincing evidence that DNA damage cannot account for observed increases in beta cell labeling with BrdU (15).

Some have used flow cytometric assessment of cell cycle stage. This requires the ability to identify beta cells among other cell types, and the availability of large numbers of cells, and is thus suited to continuously growing cell lines containing a single cell type such as rat Ins1 cells or mouse β-TC3 cells (61). Here, one can identify the percentage of cells engaged in G1/0, in S and in G2/M, but one cannot determine whether cells are successfully transitioning from one stage to another, or whether they are arrested in S or G2/M phases. The work-around here is simply performing cell counts at 24, 48, 72 hours after treatment with a putative mitogenic compound to ascertain whether actual cell numbers are increasing. Unfortunately, this approach does not lend itself to studying human beta cells, since islets are mixed cell populations, proliferate at low rates under the best circumstances, and survive in culture only a matter of days.

As outlined earlier, we have developed a method to label human beta cells using an adenovirus expressing the bright green fluorescent protein, ZsGreen, under the control of the RIP1 promoter (28). This allows labeling and detecting human beta cells by FACS among mixed human islet populations, and quantifying the numbers of beta cells in control and drug-treated samples. Using this technique, we have assessed actual human beta cell numbers in adult human islets in response to the harmine-TGFβ inhibitor combination (28) and the harmine-exenatide combination (29), and have observed clear and statistically significant increases in human beta cell numbers within 4 days of drug exposure (**Figure 6**). We have employed several controls in these studies, including: fluorescent microspheres as loading controls and recovery standards for FACS; studying a range of human islet doses; treating human islets with cytokines to show that we can use this technique to quantify beta cell death and loss; avoiding possible issues with adenoviral effects on beta cell health or counting, through the use of an independent stable human ES cell line in which GFP is knocked into the insulin locus, prior to treatment with the harmine-TGFβ inhibitor combination and the harmine-exenatide combination.

Thus, overall we are confident that harmine alone, the harmine-TGFβ inhibitor combination and the harmine-exenatide combination increase actual numbers of human beta cells based on marked increases in the Ki67, BrdU and PHH3 immunolabeling, an absence of increases in markers of cell death or DNA damage, and reproducible and statistically significant increases in the numbers of human beta cells using the FACS-based method in the preceding paragraph (28, 29). On the other hand, since the studies described above were performed *in vitro*, it would be even better to be able to assess human beta cell mass increase *in vivo* over the long term. This will need to await imaging tools that are able to assess beta cell mass *in vivo*.

### How Much Beta Cell Proliferation Is Enough?

Human beta proliferation rates (Ki67 labeling indices) in the first year of life average ~2% (13, 26, 27). Thus, one might argue that this is the “peak physiologic rate” of human beta cell proliferation, and that 2-3% per day should be sufficient to repopulate the pancreas in T1D and T2D as long as there are residual beta cells to serve as the “starting material”. On the other hand, we have observed that rates of human beta cell proliferation in dispersed human islets *in vitro* are higher than in transplanted human islets in immunocompromised mouse models. For example, harmine alone, harmine-TGFβ inhibitor combination and the harmine-exenatide combination generated Ki67 labeling average indices *in vitro* in tissue culture of ~2%, 5-8% and 5-7%, respectively, but considerably lower rates in human islet grafts *in vivo*: 0.6%, 1.5% and 1.2% (28, 29, 48). The reasons behind the lower proliferative rates *in vivo* as compared to *in vitro* settings are uncertain, but may relate to different drug dose exposures or pharmacokinetics *in vivo vs*. *in vitro*, repression of proliferation *in vivo* by suppressive factors derived from adjacent normal tissues, and/or other unknown factors. From the *in vivo* data, one might reasonably argue that higher rates of proliferation, and more potent beta cell mitogenic drugs, will be needed to return beta cell numbers from the low residual quantities in late T1D and in T2D to normal. If the enhanced beta cell differentiation observed *in vitro* applies *in vivo* (28, 29, 48), this also would benefit both T1D and T2D. Of course, the answer to this question will remain unknown until the drugs are tested in humans, but the fact that harmine alone and the harmine-exenatide combination can enhance human insulin secretion and attenuate or reverse diabetes in diabetic immunodeficient mice holds promise (29). In the meantime, development of sensitive and specific tools to allow imaging of human beta cells in immunodeficient mice and in humans will aid in these pursuits.

### Reasons for Human Beta Cell Recalcitrance to Replication: Quiescence, Senescence and Terminal Differentiation

**Figure 9:**
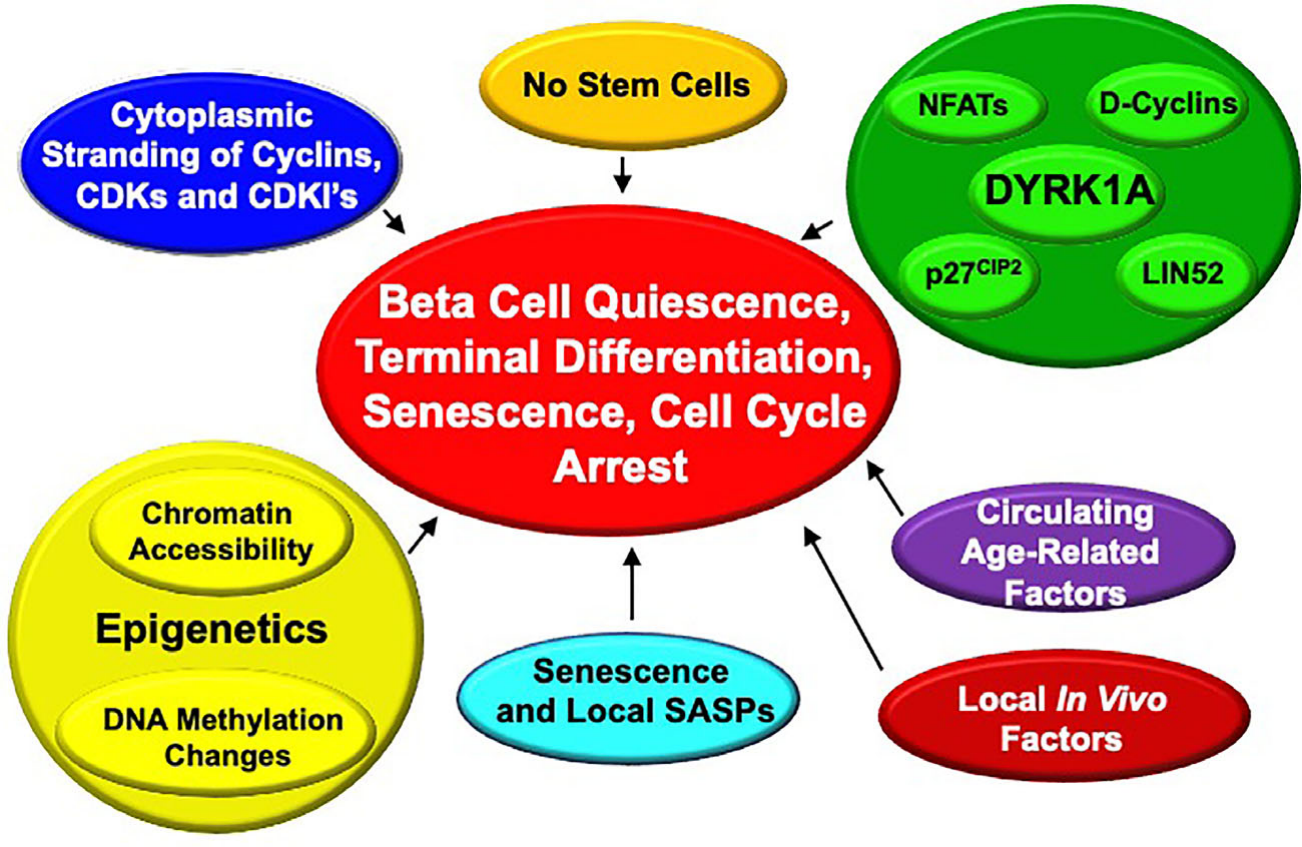
Proposed Mechanisms for Enforced “Quiescence”, “Terminal Differentiation” or “Senescence” in Adult Human Beta Cells. See text for details.

It is clear that adult human beta cells are refractory to induction of proliferation, as summarized earlier: basal labeling indices *in vitro* and *in vivo* are in the 0.0-0.5% range as assessed using Ki67, PCNA, and 18-24 hours of BrdU or EdU exposure (13, 26–29, 33, 34, 45, 47–55, 62). While harmine and other DYRK1A inhibitors increase proliferation, this is only in the 2-4% range (28, 47–55), and DYRK1A inhibitors in combination treatment with GLP1 receptor agonists or TGFβ inhibitors increase this only to 5-8% (28, 29). This means that even under optimal circumstances, >90% of beta cells do not proliferate. This resistance to proliferation has been variably attributed to “terminal differentiation”, “quiescence” and/or “senescence”, but what these terms mean in molecular or mechanistic terms for beta cells remains speculative. We summarize potential mechanisms in **Figure 9**.

In our own work, we have observed that most cell cycle molecules (cyclins, CDKs, cell cycle inhibitors, etc.) are located in the cytoplasm of beta cells where they would be predicted to be unable to drive cell cycle entry, but some can enter the nucleus when overexpressed at high levels (63, 64). Why and how this restriction to the cytoplasm occurs, and if it is a critical control point for beta cell cycle entry remains uncertain.

Avrahami, Kaestner et al. have performed an extensive analysis genome-wide DNA methylation of young (4-6 week old) and aged (16-20 months old) mice (65). They observed a general drift in genome-wide methylation, with a specific and greater increase in methylation of putative enhancers related to pro-cell cycle genes (for example, *Ki67* itself, *Ccnd3* encoding cyclin D3, *Plk1* encoding the mitosis-related gene polo-like kinase 1) and a decrease in methylation in putative enhancers of the cell cycle inhibitor gene, *cdkn2a* encoding p16^INK4^. These findings are consistent with a model in which age-related changes in DNA methylation occur that favor expression of cell cycle inhibitors and reduce expression of cell cycle activating genes. Notably, a similar pattern was observed in expression of human islets for *CDK6* (reduced) and *CDKN2A* (increased) in islets from adult *vs*. childhood organ donors (66). Thus, age-related alterations in enhancer methylation of cell cycle genes would appear to contribute to some degree in adult human and mouse beta cell resistance to proliferation.

These findings complement chromatin epigenetic data in mice suggesting that repressive histone marks (H3K27me3) written by Polycomb Complex genes such as BMI1 and EZH2, and activating histone marks (H3K4me3) written by Trithorax members likely contribute to control of juvenile *vs* adult beta cell proliferation in an age-related manner (28, 60, 67–74). Along these lines, we have shown that combined treatment with harmine and TGFβ inhibitors appears to disrupt binding of SMADs and the Trithorax members MEN1 and KDM6A to regulatory regions of *CDKN1C* encoding p57^KIP2^ and *CDKN1A* encoding p21^CIP1^, thereby relieving repression of cell cycle progression in the beta cell (28).

Bonner-Weir and Bhushan have suggested that beta cell senescence, driven by, or associated with, increased expression of senescence markers p16^INK4^, senescence-associated beta-galactosidase and secretion of senescence associated secretory proteins (SASPs) also plays an important role in maintaining cell cycle arrest, and in beta cell dysfunction with age (74, 75).

Of course, the repeated observation that DYRK1A inhibitors enable quiescent adult human beta cells to proliferate, suggests that age-related expression, function, subcellular localization of DYRK1A may play a role in age-related changes in beta cell proliferation. This remains an open question; in our limited RNAseq datasets comparing adult to juvenile whole islets, we have not observed an age-related difference in DYRK1A or DYRK1B expression. This may be an interesting area to pursue in future studies.

In a remarkable set of parabiosis experiments, Dor et al. have shown that joining young to old mice leads to induction of proliferation in previously quiescent beta cells of the old mice (76). Conversely, beta cells in islets from young mice transplanted into old mice proliferated at lower rates than those transplanted into young mice (76). This suggests that circulating factors that suppress beta cell proliferation exist in old mice, or that circulating factors that promote proliferation are present in juvenile mouse serum, but absent in old mice.

Finally, as noted in the preceding section (*“How Much Beta Cell Proliferation Is Enough?”*), human beta cell proliferation rates *in vitro* are substantially higher than *in vivo* in human islets transplanted into immunodeficient mice. We speculate that this may be due to local production of uncertain proliferation-inhibiting factors such as TGFβ superfamily members by host cells in the graft site, or contact inhibition mediated *via* integrins, focal adhesion kinases, connexins, etc, but have no data to support this possibility.

The bottom line here is that the systemic and cellular mechanisms that enforce cell cycle arrest in adult human beta cells are complex and incompletely understood, and the possibilities listed above likely inter-related. We believe that this area provides fertile ground for future beta cell regenerative drug discovery.

### Are There Additional Mechanism(s) of Action of DYRK1A Inhibitors?

Although the calcineurin-NFaT model described earlier seems well supported, there may well be additional mechanisms of action through which DYRK1A inhibitors drive proliferation in beta cells and in other cell types. For example, DYRK1A has been reported to phosphorylate a number of substrates in addition to NFaTs, exemplified by Thr^212^ in Tau protein (77), and Thr in amyloid precursor protein (APP) (78), both associated with Alzheimer disease, and RNA polymerase (79). DYRK1A phosphorylates Thr^125^ in caspase 9, apparently enhancing cell survival (80). Li et al. have suggested that DYRK1A can also serve as a transcription factor, through interaction with the histone acetylase, EP300, and the transcriptional co-activator CREBBP, also known as CBP (81). Among cell cycle control molecules, DYRK1A is reported to phosphorylate Ser^15^ in p53, enhancing its activity, and inducing expression of downstream targets such as p21^CIP1^ (82). DYRK1A also can phosphorylate cyclin D3 and cyclin D1 at Thr^283^, leading to their accelerated degradation, actions that again would favor cell cycle arrest (83, 84). In the context of beta cells, Annes has suggested that DYRK1A can phosphorylate the DREAM complex member, LIN52, at Ser^28^, as well as the cell cycle inhibitor, p27^CIP1^, at Ser^10^, enhancing its stability (53). Dirice et al. observed that 5-IT reduces Ser^10^ phosphorylation in p27^CIP1^ (50). The predicted net effects of these LIN52, p27^CIP1^, p53, and D-cyclin phosphorylations all would be cell cycle arrest. Conversely, DYRK1A inhibition or loss would be predicted to favor cell cycle entry. Collectively, these observations suggest the possibility that DYRK1A inhibitors such as harmine may induce human beta cell proliferation through multiple pathways, in addition to the canonical inhibition of the nuclear actions of NFaTs. Determining if any or all of these additional pathways apply to human beta cells, and to what degree, will be of great interest.

### How Do DYRK1A Inhibitors Enhance Human Beta Cell Differentiation?

We noted earlier in “Areas of Consensus”, that harmine not only drives human beta cell proliferation, it also appears to enhance human beta cell differentiation *in vitro* and function *in vivo* (28, 29, 48). These findings may seem counterintuitive, since one might have expected that proliferating beta cells would de-differentiate. We interpret this to mean that transient de-differentiation likely occurs in the minority of beta cells during the ~24 hours of cell cycle transit, whereas the non-cycling majority of beta cells increase expression of the differentiation markers described earlier. The cellular mechanisms through which DYRK1A inhibitors drive beta cell differentiation are uncertain. Some available evidence would suggest that these effects may be due to DYRK1A inhibition and enhanced nuclear NFaT transcription of the genes listed above. More specifically, Goodyer and Kim have shown that NFaTs bind to, and are required for transcription of beta cell cycle genes (Cyclin A, FOXM1, Cyclin D2), and genes required for beta cell differentiation and function (insulin, PDX1, SLC2A2, GK, IAPP and chromogranins A and B) in mouse islets, and in some instances in human islets (31). Whether this is a feature of harmine and 5-IT alone or of all DYRK1A inhibitors, whether NFaTs can induce these effects in human beta cells, and among the NFaT family, which one(s) is(are) responsible are unknown. It is also unknown whether these effects can be ascribed with certainty to DYRK1A inhibition, or whether they may be mediated by additional, currently unknown targets of harmine. Since beta cells are de-differentiated in people with both T1D and T2D, identifying drugs that enhance both proliferation as well as differentiation, as well as their full mechanism(s) of action is an important future challenge.

### Will There Be Additional Classes of Human Beta Cell Proliferative Drugs?

Until recently, there were no human beta cell regenerative molecules that generated rates of proliferation sufficient to replenish beta cell mass in humans with T1D or T2D. The scenario has clearly changed over the past five years. This raises the question, will additional candidates appear? We suspect the answer to this is likely “yes” for several reasons. First, as reviewed above, we are still in the early days of understanding the mechanisms that regulate beta cell cycle arrest and quiescence; thus, it is not difficult to imagine that advances in this area will point to new, previously unanticipated targets.

Second, we know from work we have done with human insulinomas (60, 85) - rare, benign proliferative tumors of the beta cell that overproduce insulin and cause hypoglycemia - that many different types of gene variants (single and multiple nucleotide mutations, copy number loss, copy number gain, chromosomal rearrangements, etc.) are associated with human beta cell proliferation in a manner that expands beta cell mass, while also preserving beta cell function (insulin production) so effectively that it leads to hypoglycemia. We believe human insulinomas contain a drug-discovery “roadmap” or “recipe” for identifying beta cell regenerative drugs. As one illustration, although most insulinomas display a distinct set of mutations or “variants”, the large majority of these occur in genes that regulate 3-D chromatin structure, and many of these are in the same families of genes that maintain cell cycle arrest alluded to earlier (60, 85). As an example, the majority of insulinomas have mutations in members of the Trithorax and/or Polycomb proteins that “write” or “erase” the epigenetic marks H3K27me3 and/or H3K4me3 and/or H3K27Ac that determine whether chromatin in the region of a specific gene is open and accessible to relevant transcription factors, or closed and inaccessible. Included in these transcriptional networks are the downstream TGFβ signaling family, the SMADs. Thus, the number one Gene Ontology bioinformatic cluster identified among the many different mutations in insulinoma included Trithorax genes such as *MEN1* and *KDM6A* along with *SMAD* genes (60). This insight accurately predicted that we might further augment the beta cell proliferation derived from DYRK1A inhibitors by adding TGFβ inhibitors to disrupt SMAD-Trithorax and Polycomb interactions (28). As an additional example, the *CREBBP* gene displays recurrent mutations or loss of copy number in insulinomas (28). *CREBBP* is in this same Trithorax gene ontology cluster with *MEN1* and *KDM6A*. This is of particular interest, because CREBBP is downstream of cAMP signaling, in partnership with the transcription factor CREB, and their co-activator EP300. The combined harmine-GLP1R agonist story described above requires GLP1R activation of cAMP signaling, which we have shown is likely mediated by CREB-CREBBP family signaling (29). Thus, human insulinomas correctly predicted both SMAD and cAMP signaling might partner with DYRK1A inhibition to enhance human beta cell proliferation. We suspect that these examples are only the tip of the iceberg, and that additional interactional therapeutic concepts buried in human insulinomas will be revealed as these studies continue.

Third, these are still early days in human beta cell regenerative drug discovery. Until recently, achieving this goal was widely viewed as impossible and not worthy of the effort involved in trying. Now that it is clearly possible, we suspect that additional interest in pharma, biotech and academia will yield additional beta cell regenerative compounds.

### DYRK1A Inhibitor Effects on Human Beta Cell Survival

DYRK1A inhibitors do not induce beta cell death or DNA damage in human islets. More specifically, assays of beta cell death (TUNEL assay) or DNA damage (γH2AX) are repeatedly negative in human beta cells treated with harmine or other DYRK1A inhibitors in settings where beta cell proliferation is occurring (28, 29, 48).

Although preventing or reducing ongoing beta cell death or loss is an important therapeutic goal, it is equally clear that simply blocking cell death alone will not reverse established T1D or T2D diabetes. For example, arresting beta cell autoimmunity with immunomodulatory therapies or glucocorticoids may delay progression of established T1D, but does not reverse diabetes, presumably reflecting the inability of adult human beta cells to regenerate spontaneously (18, 25, 86). This is in part the rationale for designing early intervention clinical trials in newly diagnosed T1D (18, 25). Thus, the important concept is that if and when effective immunomodulatory therapy emerges for T1D, or re-differentiation therapies emerge for T2D, human beta cell regenerative therapies, such as DYRK1A inhibitors, will be required to replenish beta cell mass in established T1D and T2D.

There may be room for cautious optimism here. As one example, DYRK1A inhibition may benefit both autoimmunity and beta cell regeneration in T1D. NFaTs were described originally in T-lymphocytes (they are Nuclear Factors activated in T-cells). As noted above, Khor et al. have suggested that harmine enhances T-reg lymphocyte differentiation and function while inhibiting pro-inflammatory Th-1 and Th-17 T-cell differentiation, actions expected to inhibit autoimmunity (39, 87, 88) How this story unfolds in the coming few years will be of great interest. As a second example, GLP1 receptor agonists including GLP1 itself and lactogenic hormones such as prolactin and placental lactogen have been shown to enhance human beta cell survival *in vitro* (35, 62, 89, 90). This may contribute to the beneficial effects of the harmine-exenatide combination. As a third example, widely used calcium channel blockers, exemplified by verapamil, enhances survival of human beta cells and are in clinical trials for T1D (91). As a fourth example, a small molecule inhibitor of thioredoxin-interacting protein (TIXNIP) called SRI-37330 shows promise for enhancing beta cell survival and function in human T2D (92). As a fifth example, sulfated polysaccharides such as dextran sulfate that protects beta cells, induce immune tolerance and ameliorates diabetes in early-onset type 1 diabetic NOD mice (39). Yet another example has been provided by Bhushan et al. who suggest that beta cell senescence and production of senescence-associated secretory proteins (SASPs) cause or accelerate beta cell death. This group has shown that senolytic drugs such as ABT-199 (Venetoclax) attenuate the senescent beta cell phenotype in mouse and human models of T1D (75).

To summarize, our own view is that any therapy that combines pro-survival effects with beta cell regenerative effects will be superior to one that only enhances proliferation. Thus, we can readily envision a future in which T1D and T2D therapy may include combinations of DYRK1A inhibitors with calcium channel blockers, TIXNIP inhibitors, dextran sulfate, senolytics, immunomodulatory antibodies and/or immunosuppressant small molecules.

### Is Beta Cell Regeneration Therapy Safe?

In 2021, this important question remains unanswered. This a question with many components. In this section, we address what we believe are the critical questions and issues regarding human safety surrounding DYRK1A inhibitors.

#### Historical Use of DYRK1A Inhibitors

The two principal sources of harmine in nature are a South American vine named *Banisteriopsis caapi*, from which an oral infusion or brew called Ayahuasca is made, and a Middle Eastern plant named *Peganum harmala* used in the form of incense, oral infusions and inhalants (93–99). The most thoroughly studied of these, at least in the West, is Ayahuasca. South American shamans have been using harmine-related compounds for at least 1000 years (93, 94). Ayahuasca is prepared from *Banisteriopsis caapi* vine, which contains harmine, blended with leaves of *Psychotria viridis*, which contains 5,5-dimethyl tryptamine (DMT), and can be consumed orally as a tea or infusion, inhaled by smoking or snuffed nasally as a powder. More recently, the psychoactive effects of these ethnological formulations have reached the modern world, attracting attention in the *New Yorker* and the *New York Times* as recreational drugs (98, 99). Most of the lay and scientific literature in the US relates to Ayahuasca. Traditional Ayahuasca induces visual hallucinations and a pleasant mood enhancement in optimal doses (96–99). In higher doses, nausea, vomiting, diarrhea, drowsiness and attenuated consciousness may occur. Importantly, in spite of the widespread and multi-century use, however, there are few or no reports of death or chronic disease.

Calloway et al. reported that a traditional brew and dose of Ayahuasca contained 252 mg of harmine, 35 mg of DMT, 159 mg of tetra-hydro-harmine (THH), and 30 mg of harmaline (96). Harmine and harmaline are monoamine oxidase (MAO) inhibitors, and DMT is a potent psychoactive agent, acting through serotoninergic pathways in the CNS (93, 96, 97). DMT is inactive when administered orally as a result of effective inactivation in the GI tract and liver by MAO. Peak harmine and DMT concentrations after a single oral dose of Ayahuasca occur at approximately 100 min following ingestion, with harmaline and THH peaking at 145-175 min post-ingestion. Peak concentrations were 115 ng/ml and 16 ng/ml, for harmine and DMT, respectively (96). The ability to observe DMT in the circulation accompanied by typical DMT visual and psychoactive effects, was attributed to MAO inhibition by harmine in the GI tract and liver, blocking DMT degradation, thus permitting achievement of psychoactive concentrations of DMT in the circulation and CNS. As a corollary, harmine is not believed to be involved in inducing the psychoactive effects, since *Banisteriopsis caapi* brews alone do not have the psychoactive effects of Ayahuasca. Further, the psychoactive effects of Ayahuasca are typical of those observed with parenteral DMT use, and are not observed with MAO inhibitors (91, 94). A second study by Riba et al. using a similar design, but in which the Ayahuasca contained greater amounts of DMT (57 mg) and lower amounts of harmine (94 mg), generated similar psychoactive effects with slightly lower peak DMT levels (12 ng/ml) (97). Interestingly, harmine was readily measured in the Ayahuasca, but was undetectable in plasma in this study (97). The authors surmised that it was entirely degraded by its first pass through the liver. The important point here is that both studies attributed the psychoactive effects of Ayahuasca to DMT and not to harmine.

This latter conclusion is supported, albeit weakly, by two additional studies. In the first, from 1956, ostensibly pure harmine was administered orally in doses ranging from 20-960 mg to volunteers (100). The authors indicate that “visual hallucinations might have occurred in the present study with the higher doses, the maximum oral dose being 960 mg.” This study suffers from many weaknesses including a poor description of the study protocol, of the harmine preparation and of the harmine assay employed. If it can be believed, however, it may suggest that oral doses as high as 960 mg may be tolerated in humans without adverse effects. In a second study, from 1964, very low doses (30-40 mg) of a poorly described harmine preparation were administered to patients, with no apparent adverse effects (101). Thus, the overall state of the art for harmine toxicity in humans is that its safety and optimal dosing in humans is unknown, because pure pharmaceutical grade harmine has never been administered to humans. We believe that this question should be addressed in a standard Phase 1 escalating single dose study in humans.

#### DYRK1A, Neurogenesis and Neurotoxicity

DYRK1A is encoded by the *DYRK1A* gene on chromosome 21. Down syndrome is characterized by three copies of all or part of chromosome 21. In all cases of Down syndrome, three copies of a critical region of chromosome 21 - the Down’s Critical Region - must be present (102, 103). This region encodes two genes, one of which is *DYRK1A*. Thus, all people with Down syndrome have three copies of the *DYRK1A* gene. Under- and overexpression of *Dyrk1a* in mice, or deletion of its drosophila homologue, *mnb*, lead to major brain abnormalities, exemplified by a shrunken brain (minibrain, hence the gene name *mnb* in drosophila) (104). Since humans with Down syndrome have three copies of *DYRK1A*, and since autopsy data indicate that DYRK1A protein is expressed at 50% higher levels than in normal (102, 103), one of the early goals of DYRK1A inhibitor research was developing their use in early childhood for people with Down syndrome. More recently, the knowledge that Alzheimer-associated proteins Tau and APP (discussed earlier) are DYRK1A substrates has attracted the study of DYRK1A inhibitors in neurodegenerative disease in general (77, 78). Conversely, as noted above, underexpression of Dyrk1a in mice and drosophila also has adverse effects on brain development (104–106), making the point that exactly the correct amount of DYRK1A production is essential for normal brain development. As a corollary, an error in DYRK1A inhibitor dosing might have its own adverse neurodevelopmental effects. Perhaps for this reason, DYRK1A inhibitors have not reached the clinic for children with Down Syndrome. Against this scenario, as noted above, chronic use of Ayahuasca for centuries and current widespread use in first world countries has not been reported to cause adverse long-term CNS consequences. Thus, this issue will only be clarified when preclinical and human studies are performed using GMP quality harmine.

In contrast to humans, there are ample studies on the psychoactive effects of harmine in rats and mice. Harmine has been administered to rodents in multiple studies for several weeks. For example, Mennenga et al. administered harmine at a single dose of 1 or 5 mg/kg SubQ to 17-month old Fisher 344 rats in an aging-memory study (107). Reus et al. administered harmine i.p. at a dose of 5, 10 and 15 mg/kg daily for 14 days in a Wistar rat depression model (108). Liu et al. administered harmine 10 and 20 mg/kg i.p. daily for 10 days in a C57BL6 mouse depression model (109). Although these studies were not designed to provide safety and toxicology data, the authors did perform behavioral studies and careful observation, and in each case, reported no adverse effects. Supporting the Liu dosing safety data, we also have used harmine in preclinical mouse studies exploring beta cell proliferation and regeneration. We administered harmine intraperitoneally (i.p.) for up to three weeks using doses of 1 and 10 mg/kg/day (28, 29, 48, 52). In C57BL6 and SCID mice receiving 1 mg/kg i.p., no adverse effects were observed. A single 10 mg/kg i.p. dose produced transient (5-15 min) tremor and hyperactivity, and resolved with no apparent residual effects (52). For this reason, we have used harmine in mice at doses between 1 and 10 mg/kg in subsequent studies and observed no apparent toxicity at the 10 mg/kg i.p. per day dose (28, 29, 48, 52). Notably, we have also synthesized a novel version of harmine, compound 2-2c described earlier (52). Unlike harmine, 2-2c does not interact with serotoninergic receptors used by DMT, has reduced exposure to the CNS, and therefore does not elicit psychoactive responses in mice at doses 3-fold higher (30 mg/kg i.p.) than those of harmine that do induce such responses. Taken together with the preceding sections, these observations suggest that harmine is unlikely to have meaningful adverse effects in humans at beta cell therapeutic doses, and that if it does, it can be modified through medicinal chemistry to diminish entry into the CNS. These issues may be particularly relevant in designing future studies in children and young adults with T1D.

#### Cardiovascular Safety

Briefly, the studies with orally administered Ayahuasca of Calloway and Riba described above suggest that administration of harmine in Ayahuasca to humans in doses up to 250 mg do not have important adverse effects on blood pressure or heart rate (96, 97). Again, these types of studies have never been performed in humans with pure harmine, and should be a part of an initial dosing study in humans.

#### Oncologic Safety

Since the *DYRK1A* gene is broadly expressed, and since DYRK1A inhibition activates beta cell proliferation, it is reasonable to wonder whether DYRK1A inhibitor small molecules might lead to undesired proliferation or oncogenic transformation in tissues and cells other than the beta cell. On the other hand, DYRK1A serves to inhibit proliferation in at least some situations. Surprisingly, perhaps, most of the reports on DYRK1A in the cancer literature describe the development and use of DYRK1A inhibitors as anti-cancer therapeutics (110, 111), and report that increased DYRK1A activity in cancer drives oncogenesis. As an example of the latter, Luna et al. report that DYRK1A is widely overexpressed among pancreatic adenocarcinomas, resulting in increased production of oncogenic hepatocyte growth factor, and that inhibition of DYRK1A by harmine attenuates the growth of pancreatic adenocarcinoma cells *in vitro* and *in vivo* (112). In accord with this, we have found no evidence that harmine in doses employed in beta cell regenerative studies are oncogenic in rodents or other species in the literature, although this may simply reflect a dearth of studies in this area.

We examined tissue histology and Ki-67 labeling in exocrine pancreas, liver, kidney, spleen, heart and intestine following one week of treatment with harmine (10 mg/kg/d) and exenatide (5 µg/kg/d) (29). As positive controls, abundant Ki67 immunolabeling was observed in normal intestinal crypt stem cells and in beta cells in human islet grafts and endogenous mouse beta cells. In contrast, no proliferation or histologic abnormalities were observed in the other organs, including pancreatic ductal epithelial cells. These findings indicate that at therapeutically relevant doses for human beta cells *in vivo*, the harmine-exenatide combination causes no off-target proliferation. This is reassuring, but of course needs to be studied and replicated for longer periods of time.

**Figure 10:**
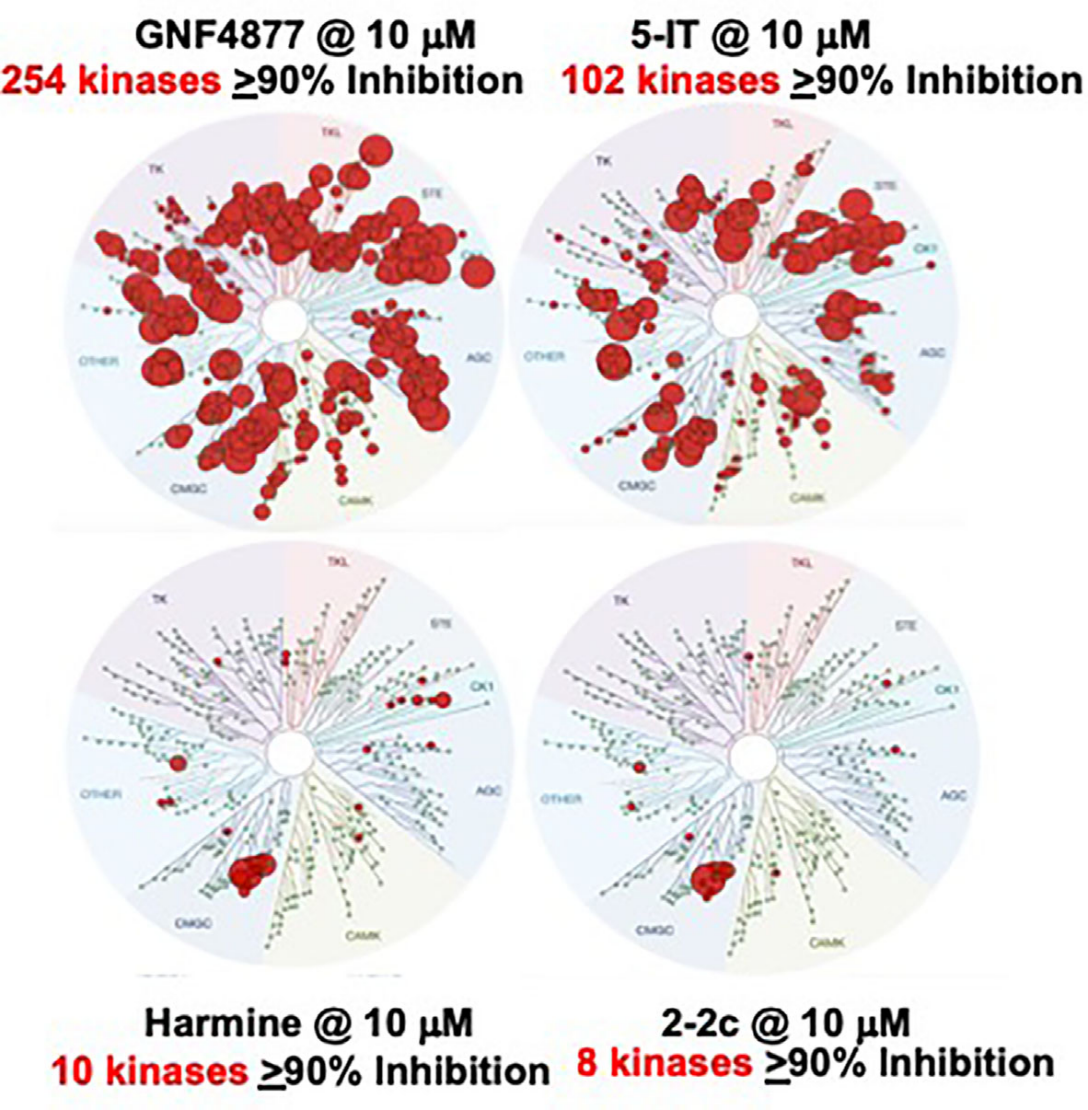
Kinome Scans for Representative Beta Cell Regenerative DYRK1A Inhibitors. GNF4877, 5-IT, harmine and Compound 2-2c were assayed for binding interactions with a standard panel of 468 human protein kinases from DiscoverX, all at the same concentration, 10 µM. Compare to **Figure 4**. GNF4877 and 5-IT are the most potent activators or human beta cell proliferation, but have poor DYRK1A specificity. GNF2133 is also a potent activator of proliferation, but has poor kinase specificity, even in a limited kinome scan (55). Among published data, compound 2-2c has the greatest human beta cell proliferation ability with the highest DYRK1A specificity. It has the additional advantage of not being able to enter the CNS, and avoids serotoninergic and adrenergic G-protein coupled receptors. Adapted from (51, 52), which contain complete details.

In contrast to our studies with harmine, a worrisome report from Liu et al. describes a novel beta cell mitogenic DYRK1A inhibitor called GNF2133 (55). At an oral dose of 100 mg/kg/d for 2 weeks in rats, GNF2133 increased Ki67 labeling in multiple organs, including exocrine pancreas, kidney, liver and heart, and led to abnormal pathology in the kidney. This dose contrasts to the therapeutic doses of GNF2133 which were substantially lower (3, 10 and 30 mg/kg/d) (55), and suggests that high doses of GNF2133 may lead to undesired proliferation and adverse effects. We attribute these findings to the high *in vivo* doses selected, and the broad kinase inhibition profile of GNF2133 (**Figure 10**). For example, a kinome scan against 250 kinases (55) {in contrast to our usual 468 kinase screen (51)} at low doses {0.2 and 2 µM (55) in contrast to our usual 10 µM screens (51)} revealed that GNF2133 hits multiple kinases in addition to DYRK1A, many of which might lead to undesired proliferation. Importantly, harmine alone was effective in inducing beta cell proliferation *in vivo*, at only 10 mg/kg (48), and when combined with exenatide, at only 1 µg/kg (28, 29). It also contrasts with our report using compound 2-2c, an even more selective and potent DYRK1A inhibitor at a than harmine, which was effective *in vivo* at 1 mg/kg (52). Thus, we believe that with several existing more selective, potent DYRK1A inhibitors other than GNF2133, off-target proliferation and other adverse effects do not occur.

#### Other Off-Target Effects of Harmine: PPARγ and the Adipocyte

Potential off-target effects on the adipocyte merit additional discussion. Waki and Tontonoz identified harmine in a high-throughput screen of small molecules able to induce the adipocyte-specifying gene PPARγ (113). Harmine was shown to enhance adipocyte differentiation from precursors, and to enhance insulin sensitivity and improve glucose tolerance control in obese C57BL6/J mice without affecting body weight. In a separate report, Nie and Wu have suggested that harmine enhances adipocyte thermogenesis both *in vitro* and *in vivo* in C57BL6/J mice on a high-fat diet (114), suggesting that the beneficial effects observed by Waki and Tontonoz may be related to a reduction in accumulation of body fat. It is not clear whether these ostensibly beneficial effects might occur in human systems, whether they occur with a variety of other DYRK1A inhibitors, and whether they are due to DYRK1A inhibition, or interference in other pathways. If these beneficial effects prove to occur with all DYRK1A inhibitors, this may further enhance enthusiasm for harmine or other DYRK1A inhibition for T2D and obesity.

#### Insulinoma

The oncogenicity discussion thus far has focused on off-target effects of DYRK1A inhibition on tissues other than the beta cell. One also might reasonably be concerned with on-target oncogenicity: inducing beta cell tumors, so called insulinomas (60, 85, 115). Indeed, as noted above, we have used insulinomas to help provide a genetic roadmap or wiring diagram of pathways useful for inducing human beta cell proliferation. Several points are important there. First, although they can be malignant, human insulinomas are almost always benign, non-malignant, non-metastatic and easily cured by simple laparoscopic removal. Second, the pathways that characterize malignant transformation are reasonably well characterized (mutations in the DAXX, ATRX, MUTYH, CHEK2, BRCA2 and mTOR pathways) (116, 117) and have not been observed with DYRK1A inhibitor therapy to date. Third, gene mutations in insulinomas and other tumors are permanent and irreversible; this contrasts to small molecule DYRK1A inhibitor treatment, which is envisioned as transient for months or years until the desired endpoint is achieved. This is standard procedure with other widely used mitogenic therapies: estrogen (for menopausal symptoms), testosterone (for androgen deficiency), growth hormone (for short stature), or parathyroid hormone (for osteoporosis) using doses and durations demonstrated to be safe and effective in clinical trials.

#### Other Pancreatic Islet Cells

Several authors, including ourselves, have observed that DYRK1A inhibitors can induce proliferation (Ki67, BrdU, EdU) in human pancreatic alpha cells, delta cells and in some cases PP-cells and ductal cells (28, 29, 48, 50, 51, 118). Reported proliferation rates have varied with the method employed and laboratory, but the signal is consistent enough to warrant careful observation in future studies.

### Is Beta Cell Targeting Necessary or Possible?

Some experts assume that since DYRK1A expression is ubiquitous, DYRK1A inhibitor therapy for diabetes will require conjugation of a DYRK1A inhibitor to a beta cell-specific targeting molecule. This presumption is inherent in requests for grant applications from the National Institutes of Health in the US and the JDRF. On the other hand, the preclinical efficacy and safety studies described above with harmine alone, 2-2c alone or harmine plus exenatide suggest that targeting may be unnecessary (29, 52). Importantly, if harmine family drugs have beneficial effects on insulin sensitivity *via* adipocytes (113, 114) and autoimmunity (39, 87, 88), targeting may actually be detrimental for diabetes.

No unequivocal beta cell surface-specific molecule is known to exist, but there are two interesting possibilities. The first is a monoclonal antibody (MAb) raised by Munkonda, Sevigny et al, and further characterized by Saunders, Powers et al. to a beta cell surface ectonuclease called NTPDase-3 or ENTPD3 (119, 120). The ENTPD3 MAb is not entirely beta cell specific, however, since it has been shown to interact with chondrocytes, and fallopian tubes, yet it is the best current option. Whether it can be conjugated to DYRK1A inhibitors, internalized by beta cells, release its small molecule cargo, and avoid off target tissues remains to be defined. These are important questions that must be pursued experimentally.

Another potential beta cell-specific target is the GLP1 receptor (GLP1R). Of course, the GLP1R is expressed on beta cells, and many GLP1R agonists are in widespread clinical use (exenatide, liraglutide, albuglutide, lixisenatide, semaglutide are all examples). On the other hand, the GLP1R is also expressed in certain CNS nuclei, the heart, gastric smooth muscle, pancreatic ductal cells and a few other tissues (121–123). Having said this, the safety data described above (29, 52, 96, 97, 107–109), and the broad and safe use of GLP1R agonists around the world, suggest that combining systemic low-dose harmine with widely used exendin-4 will not lead to undesired off-target effects, while generating a promising initial safety profile.

Other possibilities exist as well. Annes et al. and Choudhary et al. have taken advantage of the presence of zinc transporters such as ZnT8 encoded by the SLC30A8 gene that allow beta cells to package insulin in dense core secretory granules, comprised of hexameric insulin crystals, the formation of which requires zinc ion (124, 125). Insulin packaging into dense core secretory granules requires crystallization of insulin hexamers. Annes et al. used a zinc-chelator, DPA, to transport GNF4877 into the mouse and human beta cell in a specific manner (124). Choudhary et al. developed a zinc chelator, ZnPD5, bearing GNF4877, as a zinc-sensitive prodrug, able to selectively release GNF4877 in the interior of beta cells (125). How these stories unfold in the upcoming months and years will be of great interest.

To summarize this section, we believe that systemic administration of harmine alone or together with a GLP1R agonist, individually or as conjugates to a GLP1R agonist or a MAb such as the ENTPD3 MAb is achievable and will likely prove to be safe. As indicated earlier, however, conjugating DYRK1A inhibitors to a beta cell-specific targeting molecule may actually be undesirable, depriving them of beneficial effects on autoimmunity and/or adipocytes. These questions can only be answered through initial safety trials in animals and humans of DYRK1A inhibitors alone, and in combination with beta cell targeting molecules.

### Beta Cell Replacement *vs*. Beta Cell Regeneration Strategies: Scalability and Cost

When one discusses human beta cell regeneration and replacement, it is generally in the context of T1D. This is in part because beta cell replacement through transplant of whole pancreas, isolated pancreatic islets or beta cells derived from human embryonic stem cells is necessarily limited by scale and cost: there are simply not enough organ donors and funding to treat the hundreds of millions of people with T1D and T2D. Organ harvesting, islet isolation and stem cell production costs are enormous. For reasons of cost and organ supply, these technologies and procedures will never be scalable to more than a few thousand people with T1D.

In contrast, the expense of orally administered harmine and other small molecule DYRK1A inhibitors is trivial in comparison, and GLP1R family drugs are already approved by regulatory agencies and in use in millions of people with T2D around the world. Thus, simply combining an oral DYRK1A inhibitor with any one of a variety of approved GLP1R agonists is achievable, cost-efficient and enormously scalable to millions of people with diabetes globally. Moreover, since the harmine-GLP1R agonist combination enhances beta cell differentiation and function, and it would be particularly attractive in the setting of T2D. Of course, as noted earlier, effective T1D treatment will require an additional feature: immunomodulatory therapy to attenuate autoimmunity, an area in which progress is being made. Thus, we envision clinical trials with DYRK1A inhibitors in people with T2D preceding those in T1D, laying the groundwork for future trials in people with T1D when effective immunomodulatory therapies are in hand.

## Summary

The past five years have witnessed a sea-change in beta cell regenerative therapies. Therapeutic human beta cell regeneration until very recently had been viewed as inconceivable and impossible, but now can reasonably be viewed as clearly possible. In this review, we have summarized this remarkable half-decade of progress. We also summarize what we believe to be the next pressing questions and hurdles to be addressed and overcome. For scientists in academia and pharma engaged in this field, it has been an exciting half-decade. For people burdened with T1D and T2D, it is clear that progress is being made; in the next half-decade we expect to see more important advances in beta cell biology and pathobiology, medicinal chemistry, T1D immunology, T2D therapy, preclinical safety studies, and early human trials with DYRK1A inhibitors.

## Author Contributions

All authors listed have made a substantial, direct, and intellectual contribution to the work, and approved it for publication.

## Funding

This work was supported by the following grants: NIH P-30 DK 020541, R01 DK105015, R-01 DK116873, R-01 DK116904, R-01 DK125285, and JDRF Grant JDRF 2-SRA-2017 514-S-B.

## Conflict of Interest

The Icahn School of Medicine at Mount Sinai has filed patents on material discussed in this manuscript on behalf of some of the authors (PW, KK, RD, AG-O, AS).

The remaining authors declare that the research was conducted in the absence of any commercial or financial relationships that could be construed as a potential conflict of interest.
